# Prevalence of metabolic syndrome and its components among rural and urban populations at a provincial hospital in Northern Rwanda: a cross-sectional study

**DOI:** 10.11604/pamj.2025.50.43.44307

**Published:** 2025-02-06

**Authors:** James Gafirita, Cuthbert Musarurwa, Evirate Ntaganda, Marguerite Uwimana, Aime Dieudonne Hirwa, Mediatrice Mukahigiro, Laetitia Twizerimana, Marie Louise Nshimirimana, Stephen Rulisa, Charlotte Bavuma, Emile Ivan, David Tumusiime

**Affiliations:** 1 School of Health Sciences, College of Medicine and Health Sciences, University of Rwanda, Kigali, Rwanda,; 2 Rwanda Biomedical Center (RBC), Ministry of Health, Kigali, Rwanda,; 3 Ruhengeri Provincial Hospital, Northern Province, Kigali, Rwanda,; 4 School of Medicine and Pharmacy, College of Medicine and Health Sciences, University of Rwanda, Kigali, Rwanda,; 5 Rwanda Food and Drug Authority (FDA), Kigali, Rwanda

**Keywords:** Prevalence, metabolic syndrome, risk factors, urban, rural, Northern Rwanda

## Abstract

**Introduction:**

Africa has long been associated with high infectious disease rates and nutritional deficiencies. However, due to lifestyle changes and nutritional transitions driven by industrialization, NCDs and over-nutrition now coexist with infectious diseases. Our study assessed the prevalence and risk factors of metabolic syndrome (MetS) and its components in rural and urban populations.

**Methods:**

this cross-sectional study enrolled participants aged 35 and 65 years presenting at a provincial hospital in Rwanda. We collected demographic, lifestyle, anthropometric, laboratory, and clinical data using the World Health Organization STEPwise tool for NCDs. We used the NCEP ATP III criteria to define the MetS criteria.

**Results:**

of the 422 study participants, the majority; 322 (76.5%) were females and the overall median (IQR) age was 47 (40-53) years. Overall, 156 (37.0%) of the participants resided in a rural area. The overall frequency of MetS was 219 (51.9%) (95% CI 47.1-56.7) and was significantly higher in participants resident in an urban area 152 (57.1%) vs 67 (42.9%) rural areas (p=0.005). Hypoalphalipoproteinaemia was the most prevalent single component of the MetS 253 (61.3%) and was also the single most prevalent component in participants from urban settings; 166 (63.6%), whilst in rural-based participants, hypertension 98 (62.8%) was the most prevalent MetS component. On multivariable logistic regression analysis, BMI, LDL-C, TC, and increased age were significantly associated with MetS in participants residing in both rural and urban areas.

**Conclusion:**

a high frequency of MetS was observed in the present study with a higher frequency occurring in urban participants. Targeted health education on behavioral risk factors is recommended.

## Introduction

The burden of non-communicable diseases (NCDs) has been progressively increasing globally. In sub-Saharan Africa (SSA), between 1990 and 2017, the proportion of disability-adjusted life years lost due to NCDs went up from 19% to 30% whereas NCDs were responsible for up to 40% of all deaths in East Africa in 2015 [1,2]. Low- and middle-income countries (LMICs) have experienced an epidemiological shift from a high burden of infectious diseases towards an increasing burden of NCDs [3]. Rapid socioeconomic growth, improved public health policies, and improved clinical management of infectious morbidities have led to increased life expectancy and an emerging threat age-related NCDs such as the metabolic syndrome (MetS) are now becoming the norm in many areas of the world [4,5].

The MetS, a cluster of metabolic disorders variably defined as the co-existence of any three of: abdominal obesity (WC), high blood pressure (HBP), elevated fasting plasma glucose (FBG), elevated serum triglycerides (TGs), and reduced levels of serum high-density lipoprotein (HDL-C) cholesterol) [6]. The co-occurrence of the multiple components of the MetS increases the risk of cardiovascular diseases (CVDs) and CVD-related morbidities and mortalities, which surpasses the risk predicted from a single component [7,8].

The prevalence of MetS is influenced by environmental factors such as urbanization, and westernization of lifestyles, characterized by physical inactivity, increased alcohol consumption, and increased atherogenic diets as well as increased prevalence of obesity in communities. Previous epidemiological studies in different geographical regions have revealed a rise in the prevalence of MetS globally, a recent systematic review and meta-analysis study in Africa revealed an overall prevalence of MetS was 32.4% (95% CI: 30.2-34.7); and was significantly higher in adults >18 years with 33.1% compared to children <18 years with 13.3%, and was significantly higher in females with 36.9% compared to males with 26.7% [9]. Whereas another systematic review and meta-analysis study on MetS in SSA reported a prevalence range of MetS range of 18.0% (95% CI: 13.3-23.3) to 11.1% (95% CI: 5.3-18.9) according to different diagnosis criteria, and prevalence was higher: in women than men, and higher in urban than rural participants [10].

Other studies elsewhere: India [11], China [12,13], Malaysia [14] have reported a higher prevalence of MetS in urban communities compared to rural-based participants. This discrepancy in geographical setting could be explained by differences in sociodemographic profiles of rural inhabitants who will most likely be leading healthier lifestyles by engaging more in physically demanding occupations. Nevertheless, studies from elsewhere in Poland [15], South Africa [16], Korea [17], and United States [18] have reported a greater prevalence of MetS among rural-based participants. The discrepancies in the rate of MetS between rural and urban inhabitants from different geographical settings could be attributed to differences in demographic and socio-cultural characteristics [19].

The World Health Organisation (WHO)-NCD Country Profile 2018, reported that NCDs accounted for 44% of all deaths in Rwanda, of which 14% were due to CVD [20]. Previous studies on NCDs and the MetS done in Rwanda have only investigated the prevalence of individual components of the MetS. A study by Ntaganda *et al*. (2022) reported a high prevalence of undiagnosed and uncontrolled hypertension in rural Rwanda [21]. Another study conducted on rural and urban-dwelling Rwandans aged 15 to 64 years reported that 1: 6 Rwandans had hyperglycaemia [22], while a cross-sectional analysis of secondary data study by Mukabutera *et al*. (2016) reported an increasing prevalence of overweight and obesity among Rwandan women which was more marked in women living in urban areas [23]. Despite the increasing burden of NCDs in SSA and Rwanda in particular, this study has holistically explored the prevalence of MetS in the Rwandan population. Furthermore, understanding the prevalence of the MetS in participants residing in areas with socioeconomic disparities can provide insights into unique risk factors and health behaviours that predispose them to NCDs thus allowing the development of the area of residence-specific health promotion and disease prevention strategies. Our study therefore determined the prevalence of MetS in a diverse Rwandan population and explored rural-urban disparities in the clustering of MetS components.

## Methods

**Study design and setting:** this cross-sectional study was carried out at the outpatient department (OPD) of the Ruhengeri Referral Teaching Hospital, Musanze District, Northern Province, in Rwanda. Data collection commenced in February 2023 and ended in April 2023. The hospital is estimated to serve a population of 400 000, from 18 health centers, and has a bed capacity of 400.

**Study population:** the study adopted the Rwandan Ministry of Health policy on non-communicable diseases screening policy that targets a population aged 35 years and above. The current study therefore recruited male and female participants aged 35 years of age and above attending the OPD at Ruhengeri Referral Teaching Hospital.

**Sample size:** a minimum sample size of 385 participants was calculated using Cochran´s formula for cross-sectional studies based on an assumed prevalence of 50%, precision of 5%, and 95% confidence interval. The sample size was inflated by a factor of 10% to cater for possible missing data. Thus, a minimum sample of 422 participants was required.

**Inclusion and exclusion criteria:** consenting consecutive participants presenting at the NCD clinic who agreed to fast for 10-14 hours before blood sample collection and aged 35-65 years were enrolled. Patients below 35 and above 65 years of age, pregnant women, and those who failed to give written informed consent.

**Data collection procedure:** the data collection procedure adopted the WHO stepwise tool for NCD surveillance [24] comprising three steps; completion of an interviewer-assisted questionnaire to collect information on sociodemographic characteristics: marital status, level of education, occupation, possession of health insurance, and occupation. Lifestyle variables were also evaluated to determine key modifiable risk factors of CVD including tobacco use, alcohol consumption, fruit and vegetable consumption, physical activity, and history of pre-existing NCDs such as high blood pressure and diabetes mellitus. Diagnosis of these NCDs was confirmed using defined clinical criteria and establishing if participants were taking prescription drugs for the treatment of the respective conditions. A diagnosis of diabetes mellitus cases was confirmed using fasting plasma glucose and glycated haemoglobin (HbA1c) levels according to the American Diabetic Association (ADA) criteria [25].

For physical measurements, height was measured in centimetres (cm) using a stadiometer (Seca, Hamburg, Germany) with the participant in an upright position and without wearing shoes. Weight was measured in kilograms (kg) using a weight measuring scale (Seca, Hamburg, Germany) with participants wearing light clothing. Waist circumference (WC) was determined in cm using normal tension measuring tape midway between the inferior angle of the ribs and the suprailiac crest. Three blood pressure (BP) measurements were taken in (mmHg) using an Omron digital automated blood pressure machine (Omron Healthcare Co., Ltd, Koto, Japan) after participants had been allowed to rest for at least five minutes. The first blood pressure reading was discarded and the mean value of the final two measurements was recorded as the participant´s blood pressure reading. Biochemical tests for plasma glucose, HbA1c, and fasting lipid profiles were carried out on all the participants. A total of 10mls whole blood was collected by venipuncture after an overnight fast (10-14 hours) and aliquoted as follows; 3mls into grey top tube, 4mls into plain tube (red top), and 3mls into purple top (EDTA). Plasma and serum were harvested from the grey top tube and the red top tube respectively after centrifugation at 3000rpm for 5-10 minutes within 30 minutes of venipuncture.

All laboratory assays, fasting plasma glucose (grey top), lipid profiles (red top), and HbA1c were carried out on the COBAS C311 auto analyzer (Roche Diagnostics, Rotkreuz, Switzerland) following the principles of good clinical laboratory practice. Briefly, the colorimetric Trinder method was used to measure plasma glucose, serum triglycerides (TG), and total cholesterol (TC). This enzymatic reaction involves a series of steps that generate hydrogen peroxide which will then react with 4-aminoantipyrine and phenol to form a colored quinoneimine. The intensity of the final color will be proportional to the concentration of the analyte in the specimen. For plasma glucose, glucose oxidase converts glucose into gluconic acid and hydrogen peroxide, whilst triglycerides are hydrolyzed by lipase resulting in the phosphorylation of glycerol with subsequent reactions generating hydrogen peroxide. Total cholesterol is determined by hydrolyzing cholesterol esters, oxidizing free cholesterol, and measuring the resulting hydrogen peroxide using the Trinder method. HDL-C was measured using a homogeneous colorimetric assay that directly measures HDL-cholesterol in serum, eliminating the need for centrifugation or precipitation steps. In this method, a polymer masks non-HDL cholesterol, preventing it from interfering with the measurement. The HDL-C then undergoes the same reactions for quantification as total cholesterol. Similarly, for the LDL-C assay, non-LDL cholesterol is masked by a polymer, preventing it from reacting with enzymes in the reagent. Low-density lipoprotein-cholesterol remains accessible and reacts with cholesterol esterase and cholesterol oxidase as described for TC. The COBAS C311 HbA1c assay is a turbidimetric immunoassay designed to accurately measure HbA1c in which red blood cells are haemolysed, releasing HbA1c into the solution which will react to specific monoclonal antibodies forming a complex. As more complexes form, they aggregate, creating turbidity in the solution. This turbidity is directly proportional to the concentration of HbA1c present.

**Socioeconomic category and education level:** study participants were stratified into five socioeconomic categories (SEC) as defined by the Rwandan government with category 5 being the wealthiest and category 1 being the poorest. The study participants were also stratified by educational level as primary/elementary school level (first 6 years of formal school starting at 7 years) followed by secondary/high school for another 6 years before enrolling for tertiary/college education.

**Metabolic syndrome criteria:** the definition of the MetS was adopted from the National Cholesterol Education Program (NCEP) Adult Treatment Panel III (NCEP ATP III) guidelines [26] which consider MetS as the presence of any three among the following five risk factors: Abdominal obesity: Defined as WC >102cm for men and >88cm for women, Elevated serum triglycerides (TG): >1.7 mmol/L or specific treatment for hypertriglyceridaemia, decreased HDL-C: <1.03 mmol/L for men or <1.29 mmol/L for women or treatment for hyperalphalipoproteinaemia; Elevated Blood Pressure (BP) >130/85 mmHg or treatment of previously diagnosed hypertension, elevated fasting glucose >5.6 mmol/L or treatment of previously diagnosed diabetes mellitus.

**Urban and rural settings:** the urban and rural settings were defined according to the 'Bureau Central des Recensements et des Etudes de Population' (BUCREP) criteria. The urban area is characterized by a high population density, mainly composed of civil servants, businessmen, and students, with important infrastructure, and a high level of urbanization. In contrast, in a rural area, there is a sparse population, with residents for the most part obtaining their income from agriculture, fishing, and livestock [27].

**Data management and analysis:** data were checked for completeness and consistency in the hard copy format and double-entered into a password-protected Excel database before being exported to Stata statistical software (Stata version 15 statistical software (StataCorp, College Station, Texas, USA). Categorical data were summarised as count and proportion n(%) whilst parametric numerical data were summarised as mean±standard deviation (SD). Non-parametric data were summarised as median and interquartile range (IQR). The Student´s t-test for independent samples was used to compare two means derived from two independent groups. Two group median comparisons of non-parametric data were achieved through the use of the Wilcoxon ranksum test. Logistic regression analysis was used to evaluate the determinants of the metabolic syndrome. Bivariate logistic regression was used in the first instance to identify putative risk factors associated with the MetS. A multivariable logistic regression model was fit incorporating variables identified from bivariate logistic regression as having a p-value of 0.25 or less for overall, urban, and rural participants separately. Logistic regression analysis results were reported as odds ratios and 95% confidence intervals [OR (95% CI)] and p-values. In all cases of statistical comparisons, the level of significance was set at p=0.05.

**Ethical approval:** the protocol was reviewed and approved by the Rwanda National Ethics Committee (32/RNEC/2022). Each participant gave written informed consent before enrolment. Participant data were anonymized by assignment of unique participant identifiers.

## Results

**Participants demographic data:** of the 422 study participants enrolled, the majority; 76.5% (n=322 were females and the overall median (IQR) age was 47(40-53) and the age range was 35-65 years. There were 156 (37.0%) participants who stated their usual area of residence as rural. There were no significant differences in gender, and median age when participants were stratified by area of residence. However, there were statistically significant differences in educational attainment (p<0.001), employment status (p<0.001), and socioeconomic category (p<0.001) with more people residing in urban areas having attained a higher level of education and in formal employment. The majority of participants residing in an urban area were of significantly higher socioeconomic status compared to participants residing in rural areas. The results of social demographic data are presented in Table 1.

**Table 1:** participants demographic data

		Area of residence		
Variable*	All participants	Urban	Rural	P-value
	n=422	n=266	n=156	
Age (years) median (IQR)	47(40-53)	47(40-53)	47.5(41-53.5	0.491
Size of the family median (IQR)	5(4-7)	5(4-6)	6(4-7)	0.065
**Gender**				
Female	322(76.5)	206(77.7)	116(74.4)	0.430
Male	99(23.5)	59(22.3)	40(25.6)	
**Age category**				
35-44 years	172(41.1)	110(41.8)	62(39.7)	0.907
45-54 years	159(37.9)	99(37.6)	60(37.7)	
>55 years	88(21.0)	54(20.5)	34(21.8)	
**Education level**				
Primary	134(32.1)	65(24.9)	69(44.2)	<0.001
Secondary	110(26.4)	72(27.6)	38(24.4)	
Tertiary	131(31.4)	108(41.4)	23(14.7)	
No formal education	42(10.1)	16(6.1)	26(16.7)	
**Employment status**				
Self-employed	205(48.8)	101(38.3)	104(66.7)	<0.001
Unemployed	49(11.6)	28(10.6)	21(13.5)	
Civil service/NGO	159(37.9)	129(48.9)	30(19.2)	
Retired	7(1.7)	6(2.3)	1(0.7)	
**Socioeconomic category**				
1	26(6.2)	6(2.3)	20(13.0)	<0.001
2	92(21.9)	45(16.9)	47(30.5)	
3	302(71.7)	214(80.5)	88(56.8)	
4	1(0.2)	1(0.4)	0	

* All n (%) unless specified; IQR: interquartile range; NGO: non-governmental organisation; Fisher’s exact Chi-square used to compare categorical variables and the Wilcoxon Ranksum test used to compare age by gender and size of family. α set at 0.05 for all statistical comparisons

**Prevalence of individual and multiple components of metabolic syndrome:** the overall prevalence of MetS was 51.9% (n=219) (95% confidence interval (CI): 47.1-56.7) and the prevalence was significantly higher (p=0.005) in participants residing in an urban area 57.1% (n=152 (95% CI: 51.2-63.1) compared to those living in rural areas 42.9% (n=67) (95% CI: 35.1 -50.7). Overall, hypoalphalipoproteinaemia was the most prevalent component of the MetS (61.3%, n=253) whilst in rural-based participants, hypertension, 62.8% (n=98) was the most prevalent component and hypoalphalipoproteinaemia was also most prevalent in the urban-based participants, 63.6% (n=166). Abdominal obesity (p=0.001) and hyperglycemia (p=0.002) were significantly more prevalent in the urban-based participants compared to their rural counterparts. Hypertriglyceridaemia was the least prevalent component overall and also in the two groups (31.0%). The details of MetS and its components are summarized in Table 2.

**Table 2:** prevalence of individual components of the MetS by area of residence

		Area of residence		
MetS category	All	Rural	urban	p-value
	n=422	n=156	n=266	
	n (%)			
**Hypertriglyceridaemia** Tg≥150mmol/Ll	130(31.4)	47(31.1)	83(31.6)	0.817
**Abdominal obesity by WC**	233(55.6)	70(45.2)	163(61.7)	0.001
Males>102cm				
Females>88cm				
**Hypoalphalipoproteinaemia**	253(61.3)	87(57.2)	166(63.6)	0.179
Males <1.03 mmol/L				
Females<1.29mmol/L				
**Hypertension**	252(59.7)	98(62.8)	154(57.9)	0.319
SBP >130 and/or DBP >85 mmHg				
**Hyperglycaemia**	207(50.0)	61(40.4)	146(55.5)	0.002
FPG>100mg/dl				
**Metabolic syndrome**				
No	203(48.1)	89(57.1)	114(42.9)	0.005
Yes	219(51.9)	67(42.9)	152(57.1)	

**Key:** FPG: fasting plasma glucose; SBP: systolic blood pressure; DBP: diastolic blood pressure; WC: waist circumference; Tg: triglycerides. Proportions are compared using the Z-test for proportions. α set at 0.05 for all statistical comparisons

**Figure 1:**
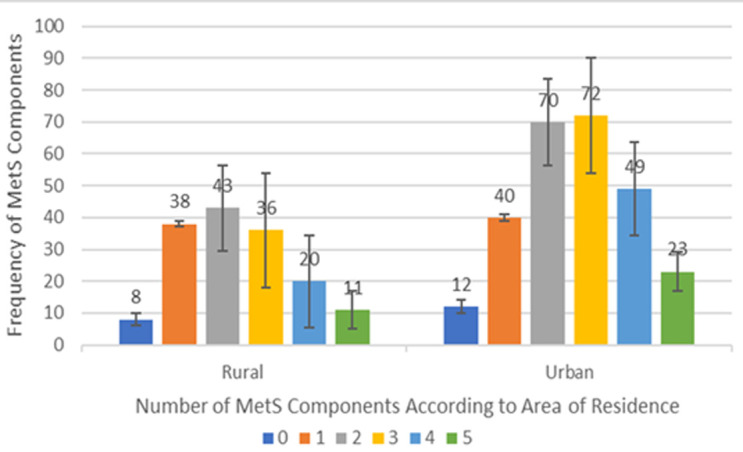
clustering of combinations of metabolic syndrome components by area of residence

The aggregate number of MetS components (hypertriglyceridaemia, hypertension, hyperglycaemia, abdominal obesity, and hypoalphalipoproteinaemia) occurring singly or together was determined based on the area of residence giving rise to six groups; (Figure 1) no MetS (0), only one component (1), two components (2), three components (3), four components (4) and all five components (5). For both groups, participants without any component of the MetS had the least frequency. Among those meeting the criteria of the MetS, overall those having all five components of the MetS were least frequent whilst those with the minimum number of components (three) defining the MetS had the highest frequency.

The socio-clinical characteristics of the study participants were compared by the area of residence participants. The results are presented in Table 3. Significantly more participants living the rural areas smoked cigarettes (p=0.018) and drank alcohol (p<0.001) compared to those residing in urban areas. Statistically significant differences were also observed in the proportions of participants who regularly consumed vegetables (p=0.002) with significantly more urban residents consuming vegetables daily whilst significantly more rural area residents regularly engaged in physically demanding occupations (p<0.001).

**Table 3:** summary of socioclinical data by area of residence

Variable	All participants (n=422)	Urban (n=266)	Rural (n=156)	p-value
Total sedentary hours/week median (IQR)	5(3-6)	5(3-7)	4(3-6)	0.185
HIV infected	17(5.1)	11(5.6)	6(4.5)	0.673
**NCD status**				
No	236(56.5)	159(60.2)	77(50.0))	0.042
Yes	182(43.5)	105(39.8)	77(50.0)	
**Ever diagnosed with hypertension?**				
No	252(59.7)	112(42.1)	58(37.2)	0.319
Yes	170(40.3)	154(57.9)	98(62.8)	
**Currently on conventional anti-hypertension medicine**				
No	14(8.5)	5(5.2)	9(13.2)	0.070
Yes	150(91.5)	91(94.8)	59(86.8)	
**Ever diagnosed with diabetes mellitus**				
No	297(72.3)	192(74.4)	105(68.6)	0.205
Yes	114(27.7)	66(25.6)	48(31.4)	
**Current cigarette smoker**				
No	385(92.3)	249(94.7)	136(88.3)	0.018
Yes	32(7.7)	14(5.3)	18(11.7)	
**Current alcohol consumption**				
No	186(45.5)	137(53.5)	49(32.0)	<0.001
Yes	223(54.5)	119(46.5)	104(68.0)	
**Consumption of vegetables**				
Rarely	10(2.4)	6(2.3)	4(2.6)	0.002
Once a week	7(1.7)	5(1.9)	2(1.3)	
2 days/week	68(16.2)	36(13.6)	32(20.7)	
3-4 days/week	164(39.0)	91(34.3)	73(47.1)	
Daily	171(40.7)	127(47.9)	44(28.4)	
**Physically demanding occupation**				
No	233(56.0)	174(66.2)	59(38.6)	<0.001
Yes	183(44.0)	89(33.8)	94(61.4)	
**Do you walk or cycle regularly**				
No	52(12.4)	32(12.1)	20(13.0)	0.796
Yes	366(87.6)	232(87.9)	134(87.0)	
**Vigorous exercise**				
No	241(60)	156(62.4)	85(55.9)	0.199
Yes	161(40)	94(37.6)	67(44.1)	

Key: *All n(%) unless specified; HIV: human immunodeficiency virus; NCD: non-communicable diseases; IQR: interquartile range. Fisher’s exact Chi-square used to compare categorical variables. α set at 0.05 for all statistical comparisons

The clinical and laboratory variables of the study participants were compared according to the participant area of residence. The results are presented in Table 4. The laboratory findings and some clinical measurements also significantly varied according to area of residence with statistically significant differences in median BMI values observed. participants residing in urban areas had a significantly higher median BMI compared to their counterparts from rural areas (p<0.001). Similarly, the median waist circumference was significantly higher in participants residing in urban centers compared to those residing in rural areas. In addition, median glycated hemoglobin levels (p=0.039) and median fasting plasma glucose levels (p=0.015) were also significantly higher in participants from urban areas compared to their rural area counterparts. There were no significant differences observed in all the lipid profile parameters when participants from urban areas were compared with participants from rural areas. The risk factors associated with the MetS were investigated using bivariate and multivariable logistic regression analysis models. The results are presented in Table 5, Table 5.1 and Table 6.

**Table 4:** comparison of demographic and laboratory findings by area of residence

		Area of residence		
Variable*	All participants; n=422	Urban; n=266	Rural; n=156	P-value
BMI kg/m^2^	27(23.1-31.2)	27.8(24.2-31.6)	25(21.6-29.7)	<0.001
Waist circumference (cm)	93(84-103)	95(88-104)	89(78-102)	0.0001
SBP (mmHg)	127.8 117-144.5)	127.5(117-144.5)	128.5(115.5-144.5)	0.838
DBP (mmHg)	81(75-90.5)	81(75-90.5)	81.5(75.8-90.8)	0.592
FPG mmol/L	5.65(4.8 – 6.7)	5.9(4.9-6.7)	5.3(4.6-6.3)	0.015
HbA1c%	5.4 (4.9 – 6.1)	5.5(5-6.1)	5.3(4.6-6.1)	0.039
TC mmol/L	4.6(4.0-5.28)	4.6(4-5.3)	4.7(3.98-5.23)	0.766
LDLC mmol/L	2.61(2.18-3.13	2.59(2.16-3.15	2.62(2.2-3.11)	0.888
HDLC mmol/L	1.14(0.98-1.34)	1.11(0.98-1.32)	1.18(0.98-1.37)	0.180
TG mmol/L	1.3(0.92 – 1.9)	1.3(0.9-1.9)	1.4(1-1.9)	0.351
Non-HDLC mmol/L	3.43(2.81-4.09)	3.44(2.83-4.11)	3.4(2.71-4.06)	0.636
LDLC/HDLC ratio	2.37(1.84-2.93)	2.37(1.85-2.97)	2.37(1.79-2.83)	0.689
TC/HDLC ratio	4.05(3.3-4.8)	4.1(3.4-4.9)	4(3.1-4.7)	0.408
TG/HDLC	1.15(0.77 – 1.79)	1.18(0.76-1.75)	1.12(0.79-1.88)	0.644

**Key:** *All values are median (interquartile range); BMI: body mass index; SBP; systolic blood pressure; DBP: diastolic blood pressure; BMI: body mass index; FBG: fasting plasma glucose; HbA1c; glycated haemoglobin; LDLC: low density lipoprotein cholesterol; HDLC: high density lipoprotein cholesterol; TG: triglycerides; TC: total cholesterol. Wilcoxan rank sum test was used to compare variables by area of residence. α set at 0.05 for all statistical comparisons

**Table 5:** bivariate logistic regression analysis of factors associated with the MetS overall and by area of residence

	Overall		Rural		Urban	
	n=422		n=156		n=266	
Variable	cOR(95%CI)	p-value	cOR (95%CI)	p-value	cOR(95%CI)	p-value
**Residence**						
Urban	Referent					
Rural	0.59(0.40-0.89)	0.012	-	-	-	-
Gender						
Female	Referent					
Male	0.26(0.16-0.43)	<0.001	0.24(0.10-0.57)	0.001	0.28(0.15-0.52)	<0.001
**Socioeconomic category**						
1	Referent		-	-	-	-
2	1.12(0.46-2.75)	0.794	0.87(0.29-2.63)	0.806	1.05(0.19-5.74)	0.959
3	1.85(0.81-4.21)	0.142	1.73(0.63-4.76)	0.286	1.26(0.25-6.40)	0.278
**Marital status**						
Married	Referent		-	-	-	-
Separated	0.76(0.33-1.76)	0.522	0.33(0.07-1.64)	0.176	1.24(0.41-3.72)	0.694
Widowed	1.85(1.06-3.23)	0.03	1.63(0.63-4.23)	0.315	1.93(0.97-3.87)	0.062
Never married	0.61(0.17-2.12)	0.434	1.73(0.63-4.76)	0.286	1.40(0.23-8.56)	0.715
**Alcohol drinking status**						
No	Referent					
Yes	0.80(0.54-1.18)	0.252	0.81(0.41-1.61)	0.552	0.92(0.56-1.51)	0.743
**Cigarette smoking status**						
No	Referent					
Yes	0.76(0.37-1.57)	0.454	1.53(0.80-4.70)	0.455	0.48(0.16-1.42)	0.186

**Table 5.1:** bivariate logistic regression analysis of factors associated with the MetS overall and by area of residence

	Overall		Rural		Urban	
	n=422		n=156		n=266	
Variable	cOR (95%CI)	p-value	cOR (95%CI)	p-value	cOR (95%CI)	p-value
**Engaged in physical work**						
No	Referent					
Yes	0.63(0.43-0.93)	0.019	1.0(0.51-1.94)	1	0.56(0.34-0.94)	0.03
**Engaged in regular walking and cycling**						
No	Referent					
Yes	0.63(0.35-1.14)	0.131	1.39(0.52-3.71)	0.508	0.35(0.15-0.82)	0.015
**Engaging in vigorous exercise**						
No	Referent					
Yes	0.80(0.53-1.19)	0.263	0.82(0.43-1.57)	0.543	0.82(0.49-1.37)	0.443
Sedentary hours	1.13(1.04-1.23)	0.005	1.10(0.94-1.27)	0.231	1.14(1.02-1.27)	0.016
BMI	1.16(1.12-1.22)	<0.001	1.23(1.13-1.33)	<0.001	1.13(1.07-1.19)	<0.001
LDLC (mmol/L)	1.61(1.26-2.05)	<0.001	1.01(1.00-1.02)	0.041	1.50(1.11-2.02)	0.008
TC mmol/L	1.48(1.24-1.78)	<0.001	1.88(1.35-2.62)	0.001	1.32(1.06-1.64)	0.013
Age (years)	1.04(1.02-1.07)	<0.001	1.02(0.98-1.06)	0.406	1.07(1.04-1.11)	<0.001

**Key:** TC: total cholesterol; BMI: body mass index; LDLC: low density lipoprotein cholesterol; BMI: body mass index

**Table 6:** multivariable logistic regression analysis of factors associated with the metabolic syndrome overall and by area of residence

	Overall n=422		Rural n=156		Urban n=266	
Variable	aOR (95%CI)	p-value	aOR (95%CI)	p-value	aOR (95%CI)	p-value
Gender	0.39(0.21-0.72)	0.003	0.29(0.09-0.89)	0.031	0.35(0.15-0.81	0.014
Age	1.06(1.03-1.10)	<0.001	1.04(0.98-1.11)	0.228	1.09(1.05-1.14)	<0.001
BMI	1.15(1.09-1.21)	<0.001	1.26(1.14-1.39)	<0.001	1.11(1.04-1.14)	0.001
Sedentary hours	0.67(0.93-1.16)	0.528	1.12(0.89-1.41)	0.342	1.03(0.90-1.18)	0.626
TC mmol/L	1.38(0.98-1.94)	0.066	2.47(1.13-5.41)	0.023	1.04(0.70-1.54)	0.864
LDLC mmol/L	0.97(0.61-1.55)	0.896	0.83(0.29-2.40)	0.734	1.20(0.69-2.09)	0.517
Walking/cycling	0.67(0.31-1.48)	0.326	1.97(0.42-9.28)	0.391	0.40(0.15-1.11)	0.078
Alcohol drinking	1.01(0.60-1.70)	0.602	1.26(0.40-3.92)	0.690	1.12(0.60-2.09)	0.734
Marital Status						
Married	Referent					
Separated	0.26(0.09-0.76)	0.014	0.03(0.002-0.4)	0.008	0.48(0.12-1.88)	0.290
Widowed	0.86(0.38-1.75)	0.608	0.83(0.18-3.69)	0.803	0.50(0.19-1.29)	0.152
Never married	0.87(0.15-4.79)	0.874	1.32(0.11-16.3)	0.829	1.04(0.12-8.76)	0.974
Socioeconomic category						
1	Referent					
2	1.12(0.39-3.21)	0.833	0.86(0.2-3.65)	0.833	1.03(0.13-7.94)	0.975
3	1.39(0.51-3.80)	0.526	0.84(0.20-3.56)	0.816	1.24(0.18-8.53)	0.825

Key: TC: total cholesterol; BMI: body mass index; LDLC: low density lipoprotein cholesterol; aOR: adjusted odds ratio

Overall, bivariate logistic regression analysis, residing in a rural area OR 0.59 (95% CI: 0.40-0.89) was inversely associated with the development of the MetS as was being male, OR 0.26 (0.16-0.43) and engaging in physically demanding work, OR 0.63 (0.43-0.93). Being male was inversely associated with MetS in both rural and urban populations whereas engaging in physically demanding work OR 0.56 (95% CI 0.34-0.94) and regularly walking or cycling, OR 0.35 (95% CI: 0.15-0.82) were inversely associated with the development of MetS only in the urban-based participants.

On the other hand, overall, being widowed, OR 1.85 (95% CI: 1.06-3.23), living a sedentary lifestyle, OR 1.13 (95% CI: 1.04-1.23), higher BMI OR: 1.16 (95% CI: 1.12-1.22), older age, OR 1.04 (95% CI: 1.02-1.07), higher serum LDLC, OR 1.61 (95% CI: 1.26-2.05) and higher serum TC, OR 1.48 (95% CI: 1.24-1.78) were positively associated with the MetS. The BMI, LDLC, and TC were similarly positively associated with MetS in participants residing in both rural and urban areas whereas older age, and leading a sedentary lifestyle were positively associated with the Mets in participants residing in urban areas only. Multivariate logistic regression analysis was further carried out by incorporating variables with p≤0.25 from bivariate logistic regression analysis (Table 6).

The results in this model revealed, overall, male gender, adjusted odds ratio (aOR) of 0.39 (95% CI: 0.21-0.72) and being separated from a spouse, aOR 0.26 (95% CI: 0.09-0.76) were significantly inversely associated with the MetS whilst being older, aOR 1.06 (95% CI: 1.03-1.10) and having a higher BMI, aOR 1.15 (95% CI: 1.09-1.21) were positively associated with the MetS. In participants residing in a rural area, male gender, aOR 0.29 (95% CI: 0.09-0.89), and being separated from a spouse, aOR 0.03 (95% CI: 0.002-0.4) were inversely associated with the MetS. Male gender was also inversely associated with the MetS in participants residing in urban areas, aOR 0.35 (95% CI: 0.15-0.81).

In participants residing in rural areas, higher BMI, aOR 1.26 (95% CI: 1.14-1.39), and higher levels of serum TC were positively associated with MetS whilst in participants living in an urban setting only being older, aOR 1.09 (95% CI: 1.05-1.14) and higher BMI, aOR 1.11 (95% CI: 1.04-1.14) were positively associated with the MetS.

## Discussion

Our study determined the burden of MetS and its components among participants residing in rural compared to urban areas. The overall prevalence of MetS was (51.9%), and was significantly higher in participants from urban areas (57.1%) compared to participants principally living in rural areas (42.9%). In bivariate logistic regression analysis (Table 5, Table 5.1), our study revealed that BMI, LDL-C, and TC were frequently associated with MetS in participants residing in both rural and urban areas, whereas older age, and leading a sedentary lifestyle were positively associated with the MetS in participants residing in urban areas only. Similarly, multivariable analyses showed that for participants residing in rural areas, higher BMI, and higher levels of serum TC were significantly associated with the MetS whilst in participants residing in an urban setting, only being older, higher BMI were significantly associated with the MetS (Table 6).

Our research findings are consistent with findings of other studies conducted elsewhere that showed a higher magnitude of MetS in participants from urban settings compared to those from rural areas settings: Faijer-Westerink *et al*. (2020) in a systematic literature review and meta-analysis that assessed the prevalence of MetS in SSA reported a high rate of MetS in urban compared rural areas [10], another recent SSA systematic review revealed a high prevalence of MetS in adults >18 years with 33.1% compared to children <18 years with 13.3% [9]. Similarly, studies from diverse geographic settings were also in concordance with our findings. Such reports include studies by Adejumo *et al*. (2017) on participants from South-West Nigeria [28], Mehata *et al*. (2018) on a nationally representative Nepalese population [29]. Another meta-analysis of studies done on Middle-Eastern populations conducted by Ansarimoghaddam *et al*. (2018) [30], as well as two studies on Iranian populations [7,31].

Contrary to our research findings, some previous studies have shown that the prevalence of MetS was higher in rural areas compared to urban areas. A study conducted in Poland revealed the prevalence of MetS in rural communities to be (40.6%) compared to (35.3%) in urban settings 14; a similar pattern of MetS was recorded in a study on the Chinese population [32], as well as a study on a Korean population by Lee *et al*. [33], and studies conducted in the United States by Trivedi *et al*. [18], and in Poland [14]. The discrepancy in the rates of MetS by area of residence in our study as cited in the literature could be due to the use of different criteria for diagnosis of MetS or the enrolment of populations that differed demographically, socially, culturally, or characterized by different socioeconomic status (SES). For instance, the present study enrolled participants aged 35-65 years, attending a healthcare facility setting while the other cited studies focused on participants aged 18 years and above, and recruited community-based participants. Advancing age is a well-documented risk factor for MetS [34], due to the various biochemical changes that occur during the aging process which affect normal human physiology. It is therefore possible that our study design that enrolled older participants (35 years) and above, and from a healthcare facility would show a higher prevalence of MetS compared to studies that enrolled younger participants.

With regards to the clustering of MetS components, our study revealed that participants recruited from the urban were more frequently associated with significantly higher median values of: WC (p=0.001), BMI (p=0.001), HB1AIC (p= 0.039), and elevated fasting plasma glucose (p=0.002) compared to their rural counterparts. These findings are in agreement with previous studies; by Lissock *et al*. [35] in a study on rural-urban difference in the prevalence of MetS components reported higher median WC and a higher proportion of participants with hyperglycaemia in urban-based participants compared to rural based participants. This could be explained in part by reduced levels of physical activity, and the substitution of indigenous African diets with energy-laden western diets by participants enrolled in urban settings [36]. In the present study, a significantly higher proportion of participants from rural areas were involved in physically demanding occupations (p<0.001) compared to participants enrolled in urban settings. The present study also observed that a significantly higher proportion of participants from rural settings smoked cigarettes (p=0.018), drank alcohol (p=0.001), and were hypertensive (62.8%) (Table 3) compared to their urban counterparts. These findings are in agreement with other studies conducted elsewhere, Poland [14], China [37] and a study by Nuotio *et al*. [38] all reported a higher magnitude of hypertension in rural-based participants compared to urban dwellers.

Rwanda´s healthcare system has made great strides in numerous areas, including accessibility to community health insurance, and curbing communicable diseases. However, the epidemiological transition has also in part been advanced by an increase in life expectancy marginally due to the pattering out of infectious diseases, and more individuals are exposed to the risk factors of NCDs through urbanization. Urbanization is associated with alterations in living habits, changes in dietary composition, physical inactivity, alcohol, and tobacco use. The epidemiological transition places NCDs as a priority in the Rwandan health care system, and thus data provided in the present study on MetS should be considered in future strategic planning to curb the NCD burden that will spur morbidity and mortality if left unchecked in Rwanda.

**The strength of the study:** our study has generated an updated prevalence of MetS in a low-income country in SSA which, similar to many other low-and middle-income countries are undergoing an epidemiological transition. The study therefore provides important baseline data for future research in similar settings. In addition, our study with a fairly large sample size is only the second study that has explored the prevalence and risk factors of MetS in Rwanda. Thirdly, the application of a mixture of assessments (biochemical, anthropometric, and self-reported sociodemographic data) gives credibility to findings from the present study. Finally, the current study extensively explored most of the risk factors associated with the MetS except for only a few that were beyond the scope of our study. These include possible genetic factors, polycystic ovary disease, and insulin resistance although aspects of the latter would be reflected by the glycaemic indices evaluated.

Our study has some limitations that include the cross-sectional nature of the study design which precludes drawing of causal associations between study variables. In addition, the study participants were predominantly female and overall restricted to those aged at least 35 years. Furthermore, all study participants were drawn from one center which was, however, a referral teaching hospital. These limitations might have introduced biases that curtail the generalisability of findings.

## Conclusion

Our study reveals an alarming prevalence of Metabolic Syndrome (MetS) in our population, driven by a complex interplay of factors. Sedentary lifestyles, unhealthy diets, socioeconomic disparities, demographic vulnerabilities, and genetic predispositions have created a perfect storm. To combat this epidemic, Rwanda must adopt a multifaceted approach. This includes promoting lifestyle modifications, addressing socioeconomic inequalities, and implementing demographic-specific strategies. Environmental considerations, such as urban planning and policy changes, also play a crucial role. By embracing a comprehensive approach, we can mitigate the MetS burden, improve population health, and reduce chronic disease risk. Our findings underscore the need for coordinated efforts to prevent, detect, and manage MetS.

**Recommendations:** policy makers from SSA countries are encouraged to develop and implement specific community-based lifestyle intervention programs tailored for both rural and urban populations. Such programs should focus on promoting healthy eating, increasing physical activity, and discouraging sedentary behavior.

### What is known about this topic

- NCDs and their associated risk factors exist in Rwanda;
- Previous studies have been published on metabolic syndrome components but no study on how these components cluster together;
- There is scarce baseline data on metabolic syndrome in Rwanda.

### What this study adds

- A high prevalence of MetS was reported in the Rwandan population, particularly in a health setting;
- Populations from urban settings are at a higher risk of developing MetS compared to their rural counterparts;
- Baseline data has been generated for the prevalence of metabolic syndrome in the Rwandan population.
